# Calibration of Speed Enforcement Down-The-Road Radars

**DOI:** 10.6028/jres.114.009

**Published:** 2009-06-01

**Authors:** John Jendzurski, Nicholas G. Paulter

**Affiliations:** Office of Law Enforcement Standards, National Institute of Standards and Technology, Gaithersburg, MD 20899

**Keywords:** calibration, down the road radar, DTR radar, radar, traffic radar, uncertainty

## Abstract

We examine the measurement uncertainty associated with different methods of calibrating the ubiquitous down-the-road (DTR) radar used in speed enforcement. These calibration methods include the use of audio frequency sources, tuning forks, a fifth wheel attached to the rear of the vehicle with the radar unit, and the speedometer of the vehicle. We also provide an analysis showing the effect of calibration uncertainty on DTR-radar speed measurement uncertainty.

## 1. Introduction

Down-the-road (DTR) radar (radio detection and ranging) is the most common type of speed measurement device used in the United States and in the rest of the world for traffic speed enforcement. There are approximately 150 000 DTR radar units in use in the United States, and almost every legal jurisdiction in the United States accepts DTR radar for evidentiary purposes in speed enforcement cases in traffic court. Consequently, the performance of these devices must be sufficient to assure the courts’ confidence in using these devices. This assurance comes from 1) compliance to established minimum performance requirements, 2) scheduled calibration by testing labs to these requirements, and 3) routine calibration in the field by the radar operator.

DTR radars are provided as either hand-held radar “guns,” dashboard-mounted units, or similar vehicle-mounted units. The DTR radar has three basic components: the transceiver/mixer head, the processing component, and the display/human interface component. DTR radars typically are found in one of two forms, one in which the display and transceiver head are combined into a common unit, and another form in which the two heads are separate but connected by an appropriate electrical interconnect. DTR radars operate within narrowly allocated (by the Federal Communications Commission) spectral bands in one of the following three broad frequency bands: X (nominally 8 GHz to 12 GHz in the United States), K (nominally 18 GHz to 26 GHz in the United States), and Ka (nominally 26 GHz to 40 GHz in the United States).

The transceiver head contains the microwave source that illuminates the target vehicle and the mixer that is used to detect the Doppler-shifted microwave signal reflected from the target vehicle. As will be discussed in the next section, the signal output from the mixer circuit (which contains a diode) is at a frequency that is the difference between the Doppler-shifted signal and the incident (illuminating) signal. This difference frequency is much lower than the frequency of the incident or the Doppler-shifted signals, and is the signal upon which a speed measurement is based. The primary purpose of the processing part of the DTR unit is to compute the speed of a target (or patrol) vehicle from the difference frequency. The display/human interface provides a readout of the computed speed, operator controls, status indicators, etc.

In this paper we are concerned with the uncertainty contributions to speed measurement using DTR radar that can be attributed to the DTR radar calibration process. This analysis does not consider uncertainties in speed measurement during actual use, that is, that are caused by operational issues with DTR radar usage.

## 2. Background

**Fig. 1:**
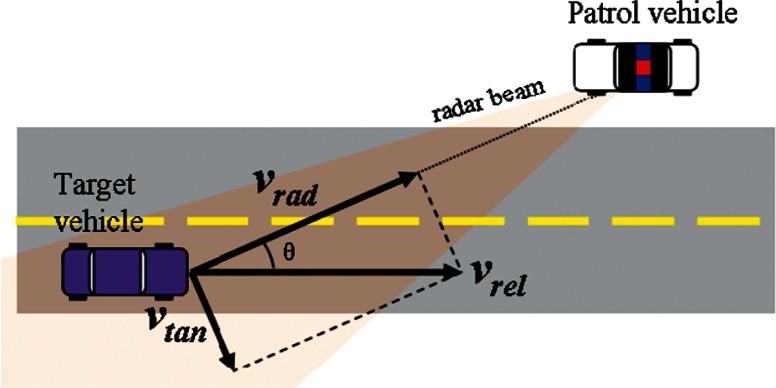
Radial and tangential components (*v_rad_* and *v_tan_*) of the relative velocity. This diagram is not drawn to scale; typical values for *θ* are on the order of a few degrees for normal DTR radar operation

As just mentioned, DTR radar uses the Doppler frequency shift, *Δf*, in the frequency of the reflected microwave signal that is used to determine speed. The value of *Δf* is dependent on *v_rad_*, which is the radial component of the relative velocity, *v_rel_*, and *f*_0_, which is the frequency of the microwave source. Figure 1 shows the relationship between *v_rel_* and its radial and tangential components, *v_rad_* and *v_tan_*. It is worth noting that *v_rad_* = *v_rel_* only when *θ* = 0, that is when the target is moving directly towards the patrol vehicle. For all other values of *θ*, *v_rad_* < *v_rel_*.

Since the DTR radar measures *v_rad_*, for the remainder of this paper, the subscript “rad” will not be explicitly shown, but it is implied that *v* is the radial velocity for all subsequent mentions and calculations.

To compute *v*, we must first have a value for *Δf*. To compute *Δf*, we start with the basic formula equating the frequency, *f*_0_, of an electromagnetic wave propagating in air to the speed of light, *c*, in air, and the wavelength, *λ*, of that wave:

$$f_{0}=\frac{c}{\lambda }.$$ (1)

The moving vehicle causes the observed wavelength to get shorter or longer, depending on whether the vehicle is moving toward the source or away from it. This change in *λ*, *Δλ*, is given by:

$$\Delta \lambda =\mp 2v\frac{1}{f_{0}}=\mp 2v\frac{\lambda_{0}}{c}$$ (2)

where the factor of 2 arises because of the round trip propagation of the microwave signal from the patrol vehicle to the target vehicle and then back to the patrol vehicle. The corresponding change in frequency, *Δf*, is given by:

$$\begin{array}{l} \Delta f=f_{D}-f_{0}=\frac{c}{\lambda_{0}\mp \Delta \lambda }-\frac{c}{\lambda_{0}}=\frac{c}{\lambda_{0}}\left(\frac{1}{1\mp 2\frac{v}{c}}-1\right)\\ \;=\frac{c}{\lambda_{0}}\left(\frac{\pm 2\frac{v}{c}}{1\mp 2\frac{v}{c}}\right)\approx \pm 2\frac{v}{\lambda_{0}}=\pm 2f_{0}\frac{v}{c}, \end{array}$$ (3)

where *f_D_* is the Doppler frequency and the approximation in (3) is valid for *v* ≪ *c*, which, hopefully, is the case for ground-based vehicles.

The Doppler shifted microwave signal, at the frequency *f_D_*, that is reflected from the target vehicle is collected by the transceiver and mixed with the source signal, at frequency *f*_0_, in a diode, to yield the signal at *Δf*. (Signal components at other frequencies are also present, but their amplitudes are much smaller than the signal at *Δf*.) To understand this mixing, we start with the well-known diode equation (see Ref. [1], page 126), which is:

$$I=I_{S}\left(e^{\frac{qV_{a}}{nkT}}-1\right)$$ (4)

where *I_S_* is the saturation current, *q* is the electronic charge, *k* is Boltzmann’s constant, *T* is the temperature in Kelvin, *n* is a constant for a given diode type, and *V_a_* is the voltage applied to the diode. In our case:

$$V_{a}=V_{r}\cos\left(2\pi f_{D}t+\theta \right)+V_{i}\cos\left(2\pi f_{0}t\right)$$ (5)

where *V_i_* is the amplitude of the local oscillator signal (LO), which in this case is the radar’s microwave source operating at *f*_0_, *V_r_* is the amplitude of the reflected signal (at *f_D_*), *t* is time, and *θ* is the phase difference between *f*_0_ and *f_D_*. The effect of mixing in a diode is more readily seen using a Taylor series expansion of the exponential term in the diode equation:

$$I=I_{S}\left(\sum_{i=0}^{\infty }{\left(\frac{q}{nkT}\right)}^{i}\frac{1}{i!}V_{a}^{i}-1\right).$$ (6)

Limiting this to first four (*i* = 0, 1, 2, 3) terms to facilitate discussion, we get:

$$I=I_{S}\left(\frac{V_{a}}{V_{B}}+\frac{1}{2}{\left[\frac{V_{a}}{V_{B}}\right]}^{2}+\frac{1}{6}{\left[\frac{V_{a}}{V_{B}}\right]}^{3}\right)$$ (7)

where $V_{B}=\frac{nkT}{q}$. It will be apparent shortly why the series was truncated. By substituting the right side of Eq. (5) for *V_a_* in Eq. (7) and expanding and combining terms, it can be shown that mixing in the diode results in frequency components at *f*_0_, *f_D_*, 2*f*_0_, 2*f_D_*, (*f*_0_ + *f_D_*), (*f*_0_ − *f_D_*), 3*f*_0_, 3*f_D_*, (2*f*_0_ + *f_D_*), (2*f_D_* + *f*_0_), (2*f*_0_ − *f_D_*), (2*f_D_* − *f*_0_), etc. The output of the mixer is filtered to eliminate all but the first-order difference frequency, (*f*_0_ − *f_D_*), which is the *Δf* computed in (3). This *Δf* is in the audio frequency range and can range from several hundred hertz to 20 kilohertz, depending on the values of *v* and *f*_0_. Present DTR radar technology and design uses a digital signal processing (DSP) unit to analyze the audio frequency signal that is output from the filter. The accuracy of the estimate of *Δf* and, consequently, the accuracy of the estimate of target vehicle speed, is dependent on the stability and known value of the DSP clock.

What is important to note here is that the computed value of *Δf* is dependent on the frequency, *f*_0_, of the microwave source, the speed of the vehicle, *v*, and the DSP clock frequency. There are also secondary effects that are dependent on *v*, the bandwidth of the audio filter, and the relative amplitude of the second-order and fourth-order difference frequencies.

## 3. Calibration Methods

There are four primary tools that are used in DTR radar calibration methods: a tuning fork, a speed simulator based on amplitude modulation (AM) of the reflected radar signal, the vehicle’s speedometer, and a fifth-wheel. A fifth wheel is a thin wheel and tire, similar in appearance to a unicycle without a seat, which is attached to the rear of the vehicle in which the radar is mounted (for a description of its usage, see Ref. [2]).

Before discussing each of these tools and associated calibration methods, it is important to note the definition of a few terms typically used to describe measurement results. The first term is measurement error. Error is simply the difference between the measured value and the actual value of the measurand, which in this case is vehicle speed. An error is not an uncertainty. The second term is measurement accuracy, which describes how close a measured value is to the actual value. Measurement error affects measurement accuracy; however, a known measurement error can be removed from a measured value to improve measurement accuracy. The last term is measurement uncertainty. Measurement uncertainty characterizes the dispersion of the values that could reasonably be attributed to the measurand. Uncertainty includes the limitations of measurement instrumentation, environmental effects, measurement standards, etc. Errors are not part of an uncertainty. However, if an error is removed from a measurement, the uncertainty in the value of the error must be considered in establishing the uncertainty of a measurand.

In the following sections, approximate values for uncertainties will be given to provide the reader an opportunity to compare different calibration methods. So that this comparison is uniform, we have arbitrarily chosen the target speed to be 96.6 km/h (60 mph). Furthermore, the uncertainties provided in all but Sec. 4 and as noted, are at the one standard deviation (1*σ*) level and represent the range in *v* corresponding to a confidence level of about 68.3 %. This means, for example, if *v* = 96.6 km/h (60 mph) and the uncertainty is 2 km/h (1.24 mph), we can say with 68.3 % confidence that the vehicle was traveling between 94.6 km/h (58.8 mph) and 98.6 km/h (61.3 mph).

### 3.1 Tuning Fork

The tuning forks used to calibrate DTR radar are usually machined from a solid aluminum blank to provide an acoustic resonant frequency that gives a specific speed indication for a given *f*_0_. To calibrate the DTR radar, the tuning fork is struck and then placed in front of the transceiver. The vibrations of the tuning fork modulate the phase of a radar signal reflected from it. This modulation simulates the difference frequency that is determined by the radar processing unit from a Doppler shifted return from a moving vehicle. However, the tuning fork does not actually simulate a moving vehicle. This difference will be clarified with the following discussion.

The tuning fork imposes a phase modulation on the incident microwave signal. The frequency, *f*_1_, of the modulation is described by:

$$f_{1}=f_{0}+A_{F}\sin\left(2\pi f_{F}t\right)$$ (8)

where *A_F_* is the amplitude of the modulation, *f_F_* is the designed resonant frequency of the tuning fork, and *t* is time. The signal reflected from the tuning fork is detected in the diode mixer and subsequently filtered to yield only the difference frequency, similar to what happens for the signal returned from the moving target vehicle (see Sec. 2). For the tuning fork modulation, this difference frequency, *Δf_F_*, is given by:

$$\Delta f_{F}=A_{F}\sin\left(2\pi f_{F}t\right).$$ (9)

Note, this frequency is sinusoidally varying with time so that the actual signal, *V_T_*, out of the filter is described by:

$$V_{T}\propto \sin\left[A_{F}\sin\left(2\pi f_{F}t\right)\right].$$ (10)

To understand how this modulation results in a signal inside the DTR radar from which the modulation frequency (and hence the speed simulated by the tuning fork) is determined, *V_T_*, must be expanded. The expansion of *V_T_* is:

$$V_{T}=2\sum_{n=1}^{\infty }J_{2n-1}\left(A_{F}\right)\cos\left[\left(2n-1\right)2\pi f_{F}t\right]$$ (11)

where the *J_i_* are Bessel functions of the first kind. This equation shows that the frequency components will be at the odd-numbered frequency harmonics of *f_F_*. Since DTR radar units are capable of measuring speeds of up to 322 km/h (200 mph), and we may want to measure speeds down to 16 km/h (10 mph), we maintain the first few Bessel functions for this discussion, which will now be determined. The Bessel functions of the first kind can be further expanded using:

$$J_{n}\left(x\right)=\sum_{k=0}^{\infty }\frac{{\left(-1\right)}^{k}{\left(\frac{x}{2}\right)}^{n+2k}}{k!\left(n+k\right)!}.$$ (12)

Because of the two factorials in the denominator and because |*x*| ≤ 1, only *J*_1_ needs to be considered (*J*_5_ ≈ 1/50 *J*_1_ or less and *J*_3_ ≈ 1/10 *J*_1_ or less). Therefore, the dominant signal component for which the DSP will compute the simulated Doppler frequency shift is at *f_F_*.

The tuning fork is machined so that *f_F_* is equal to *Δf*, for a particular pair of *v* and *f*_0_. The value of *f_F_* is dependent on the material of construction and the length and cross sectional area of the tines. Consequently, a given tuning fork should only be used to calibrate a DTR radar operating at a specified frequency and for a given speed.

The main advantages of the tuning fork calibrations are that the tuning forks are inexpensive to make, are small and portable, and require no power. They are also easy to use in the field, and this allows the officer to test the operation of the DTR radar often. However, because the material most often used is aluminum, *f_F_* will depend on temperature, which is now discussed.

**Fig. 2:**
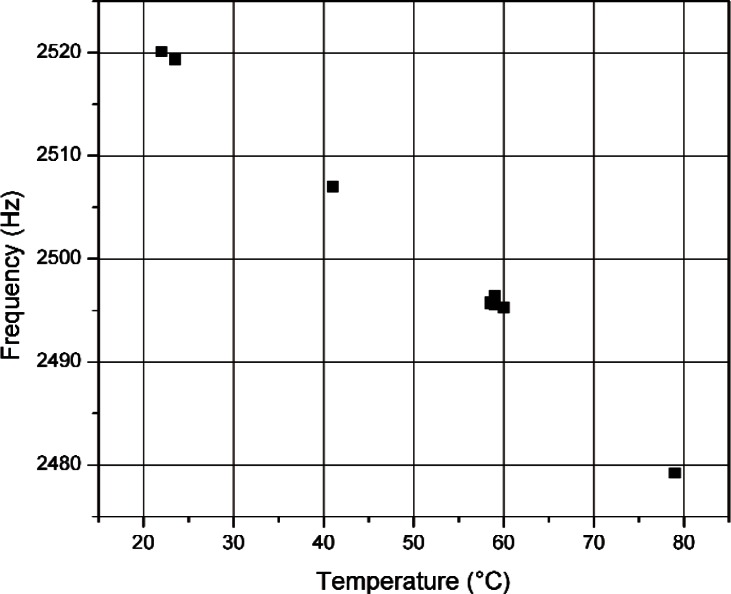
Plot of frequency (*f_F_*) vs. temperature for a tuning fork designed for 56.3 km/h (35 mph) at K-band. The Type A standard uncertainty is approximately 0.025 Hz.

An extensive test over the entire range of tuning fork operating temperatures was not performed, but *f_F_* for several tuning forks was measured over a smaller range of temperatures to demonstrate the relationship between temperature and *f_F_*. The tuning forks were placed in a temperature-controlled chamber for several hours before each measurement. Typically tuning forks are stored in the interior of a vehicle and are subject to the ambient temperature of the vehicle and possibly direct sunlight. The tuning forks were all actual forks used to calibrate DTR radar. Figure 2 shows the results of the measurements for one particular tuning fork (referred to here as TF1) designed to simulate a vehicle moving at 56.3 km/h (35 mph) for a K-band DTR radar.

A linear least squares approximation was used to obtain a formula to fit the data shown in Fig. 2 for TF1 as well as data (not shown) for the other tuning forks. The result is the following formula,

$$f_{F}=S_{TF}T+f_{TF,0}$$ (13)

where *S_TF_* is the slope, in Hz/°C, of the fit to the *f_F_* vs. *T* data and *f_TF_*,_0_ is the intercept of the fit at *T* = 0 °C. The values of *S_TF_* and *f_TF_*,_0_ are unique for each tuning fork and, for the tuning forks examined here, these values are given in Table 1. Equation (13) can be used to approximate the variation in speed measurement for a given tuning fork. For example, for TF1 and a K-band radar unit transmitting at 24.05 GHz, a temperature variation from −12.2 °C to 71.1 °C (10 °F to 160 °F) would cause a variation in *Δf* that corresponds to a target vehicle speed ranging from 55.8 km/h to 57.2 km/h (34.7 mph to 35.5 mph).

It should be noted that *f_F_* is not dependent on *f*_0_, and so the signal analyzed by the DSP is not dependent on *f*_0_. Consequently, the computed tuning-fork speed, *v_tf_*, of the tuning-fork simulated target vehicle speed is:

$$v_{tf}=\frac{c}{2}\frac{f_{F}}{f_{0}}$$ (14)

which means that the tuning fork must be used at the radar frequency, *f*_0_, for which it was designed otherwise the value of *v* will be incorrect. Similarly, drift in *f*_0_ will cause errors in *v_tf_*. To minimize the uncertainty in the value of *f_F_* and subsequently *v_tf_*, *f_F_* should be measured using a frequency counter or spectrum analyzer. In this case the standard uncertainty in *f_F_* ≈ 10^−5^ *f_F_*.

#### 3.1.1 Uncertainty in Tuning-Fork-Computed Target Vehicle Speed, $u_{v_{tf}}$

The standard uncertainty in the speed computed using a tuning fork, $u_{v_{tf}}$, is computed and presented using standard methods [2,3] to be:

$$u_{v_{tf}}=v_{tf}\sqrt{\frac{u_{f_{F}}^{2}}{f_{F}^{2}}+\frac{u_{f_{0}}^{2}}{f_{0}^{2}}}$$ (15)

where $u_{f_{0}}$ is the standard uncertainty in *f*_0_, which is about 10^−5^ *f*_0_ for typical microwave sources under normal operating conditions, and $u_{f_{F}}$ is the standard uncertainty in *f_F_*, which can be expanded using (13) to give:

$$u_{f_{F}}=\sqrt{\sigma_{f_{T=0}}^{2}+T^{2}\sigma_{S}^{2}+S_{TF}{}^{2}\sigma_{T}^{2}}$$ (16)

where $\sigma_{f_{F}}$ is the standard deviation in the measurement of *f_F_*, which is about 10^−5^ *f_F_*, $\sigma_{f_{T=0}}$ is the variation in the value of $f_{FT,0}$ in the fit to the *f_F_* vs. *T* data, *σ*_S_ is the variation (residuals) in the fit to the *f_F_* vs. *T* data, and *σ_T_* is the uncertainty in *T* (0.5 °C for this measurement). For typical values of the contributing uncertainty parameters, $u_{v_{tf}}\approx 3.1\times 10^{-3}v_{tf},$ or about 0.3 km/h (0.2 mph) for *v* = 96.6 km/h (60 mph).

### 3.2 Speed Simulator Based on Amplitude Modulation of the Reflected Signal

DTR radars may be calibrated in a laboratory using a moving target simulator. The particular simulator discussed here is a small benchtop anechoic chamber comprising a radar transceiver that receives the radar signal (at *f*_0_) and subsequently retransmits this signal to the radar under test after this signal has been amplitude modulated. To the best of our knowledge, this is the only simulator design currently used. An audio function generator provides the modulation via a PiN voltagetunable diode attenuator. As with the tuning fork, we will provide an expression describing the effect of this modulation on the signal. The amplitude modulated signal is:

$$V_{r}=V_{i}\cos\left(2\pi f_{0}t\right)A_{m}\cos\left(2\pi f_{AM}t+\varphi_{AM}\right),$$ (17)

where *A_m_* is the modulation amplitude, *f_AM_* is the modulation frequency, and *φ_AM_* is the modulation phase. Expanding this gives:

$$\begin{array}{c} V_{r}=\frac{V_{i}A_{m}}{2}\{\cos\left[2\pi t\left(f_{0}+f_{AM}\right)+\varphi_{AM}\right]\\ +\cos\left[2\pi t\left(f_{0}-f_{AM}\right)-\varphi_{AM}\right]\}. \end{array}$$ (18)

Equation (18) describes the signal that will be mixed in the diode with local oscillator (LO) signal from the radar. As we did for the tuning fork (which is a case describing phase modulation), we use the approximation to the diode equation response to find that the frequencies of the signal output from the diode mixer and filter are at ± *f_AM_*. Therefore, to calibrate a DTR radar, *f_AM_* has to equal the *Δf*. This calibration process is more versatile than using a tuning fork because *f_AM_* can be adjusted to almost any desired frequency. It should be noted that *f_AM_* is not dependent on *f*_0_, and so the signal analyzed by the DSP is not dependent on *f*_0_. The speed estimate, *v_AM_*, based on the simulator, is given by:

$$v_{AM}=\frac{c}{2}\frac{f_{AM}}{f_{0}}.$$ (19)

#### 3.2.1 Uncertainty in Simulator-Computed Target Vehicle Speed, $u_{v_{AM}}$

The uncertainty in the speed computed using a amplitude modulating moving target simulator, $u_{v_{AM}}$, is computed from (19) to be:

$$u_{v_{am}}=\frac{c}{2}\frac{f_{AM}}{f_{0}}\sqrt{\frac{u_{f_{AM}}^{2}}{f_{AM}^{2}}+\frac{u_{f_{0}}^{2}}{f_{0}^{2}},}$$ (20)

where $u_{f_{AM}}$ is the uncertainty in the frequency of the modulation, which is equal to the clock frequency uncertainty of the source, so that $u_{f_{AM}}\leq 10^{-5}f_{AM}$. Consequently $u_{v_{AM}}\approx 1.4\times 10^{-5}v_{AM}$ or about 0.0014 km/h (0.0008 mph) for *v* = 96.6 km/h (60 mph).

### 3.3 Vehicle Speedometer

The speedometer is a device that counts the number of times an object of a given radius rotates for a given time. Since the late 1980s, the most common implementation in passenger vehicles is an electronic speedometer [4]. In this design, an electronic sensor measures the number of rotations, *N*, of the transmission’s output shaft during an interval of time, *t_N_*. Accordingly, the speed obtained from the speedometer, *v_sm_*, is computed from a measure of the number, *N*, of rotations; the effective radius of the tire, *r_eff_*; the known differential gear ratio, *g_diff_*; and the time, *t_N_*.

$$v_{sm}=\frac{2\pi r_{eff}N}{g_{diff}t_{N}}.$$ (21)

*N* and *t_N_* can be determined relatively more accurately than *r_eff_* because the object is typically a pneumatic tire, and the effective radius can change due to the variety of influences that follow.

#### 3.3.1 Uncertainty in Speedometer-Computed Target Vehicle Speed, $u_{v_{sm}}$

The uncertainty in the speed computed using the vehicle’s speedometer, $u_{v_{sm}}$, using (21), is given by:

$$u_{v_{sm}}=2\pi \frac{r_{eff}}{g_{diff}}\frac{N}{t_{N}}\sqrt{\frac{u_{r_{eff}}^{2}}{r_{eff}^{2}}+\frac{u_{N}^{2}}{N^{2}}+\frac{u_{t_{N}}^{2}}{t_{N}^{2}}+\frac{u_{g_{diff}}^{2}}{g_{diff}^{2}},}$$ (22)

where $u_{t_{N}}$ is the uncertainty in the determination of the time period required for *N* revolutions, $u_{N}$ is the uncertainty in the number of revolutions counted, $u_{g_{diff}}$, is the uncertainty in the differential’s input/output gear ratio, and $u_{r_{eff}}$ is the uncertainty in the effective radius of the tire. Typically, $u_{t_{N}}$ is fixed by the uncertainties in the clock frequency, so we can approximate $u_{t_{N}}$by: $u_{t_{N}}\approx 10^{-5}f_{clk}^{-1}$. The uncertainty in *N* will be less than *N* will be less than 1 revolution, and typical values for uN=140 The value of $u_{g_{diff}}$ is, based on typical manufacture, to be about 0.005 $u_{g_{diff}}$, and in typical automotive applications 2.5 ≤ $u_{g_{diff}}$ ≤ 3.5. An approximate value for $u_{r_{eff}}$ is 1.65 cm, and the Appendix contains a discussion and derivation of this value. Using these values for the uncertainties and *g_diff_* = 3, *t_N_* = 0.5 s, and *r_eff_* = 0.326 m (typical values) for this example gives $u_{v_{sm}}\approx \sqrt{2.587\times 10^{-3}v_{sm}{}^{2}+1.165\times 10^{-3}\frac{m^{2}}{s^{2}},}$ or 4.9 km/h (3.1 mph) for *v* = 96.6 km/h (60 mph).

### 3.4 Fifth Wheel

As previously mentioned, speedometers determine speed by directly measuring the rotation of the transmission output shaft, and then applying a conversion factor to give the vehicle’s speed. The conversion factor manifests itself differently in mechanical and electronic speedometers. In mechanical speedometers it can be adjusted by changing the gearing on the speedometer cable, the strength of the permanent magnet or the stiffness of the restraining spring. In electronic speedometers, the conversion is handled by a DSP which converts pulses from the detector to a signal for display. In either case, accurately setting the conversion factor requires knowledge of the vehicle speed. The fifth wheel is towed behind the vehicle and measures the vehicle’s speed, *v*_5_*_th_*, which may be used to set the vehicle speedometer’s conversion factor

$$v_{5th}=2\pi r_{eff}\frac{N}{t_{N}}.$$ (23)

The fifth wheel is itself a calibrated tool. Calibration is performed by timing several passes at constant speed in a straight line across a calibrated distance, typically 0.5 miles. The ASTM calibration document describes the calibration technique in detail [1].

#### 3.4.1. Uncertainty in Speed Obtained From the Fifth Wheel, $u_{v_{5th}}$

Fifth wheels are immune to many of the factors that affect speedometer uncertainty on passenger vehicles. Fifth wheels are always free rolling, operate within a narrow range of normal load (vertical load), have tires which vary little with speed and are not intended for severe cornering. This reduces the number of previously mentioned parameters that can introduce errors in a speedometer measurement. Nevertheless some of the tire effects that are described in the previous section should be accounted for in the fifth wheel. The uncertainty in *v*_5_*_th_* can be approximated by:

$$u_{v_{5th}}=\sqrt{{\left(2\pi r_{eff}\frac{N}{t_{N}}\right)}^{2}\left(\frac{u_{r_{eff}}^{2}}{r_{eff}^{2}}+\frac{u_{N}^{2}}{N^{2}}+\frac{u_{t_{N}}^{2}}{t_{N}^{2}}\right)+u_{v_{5th,cal}}^{2},}$$ (24)

where $u_{v_{5th,cal}}$ is the uncertainty in the fifth wheel calibration process, which is about $5\times 10^{-3}v_{5th},$ and the remaining uncertainties are same as those described in Sec. 3.3, but as applied to the fifth wheel. The uncertainty in *r_eff_* for the fifth wheel is similar to that shown for the speedometer (see Eq. A8) but with some terms missing, as mentioned above. This uncertainty, $u_{r_{eff}},$ is given by:

$$u_{r_{eff}}=\sqrt{u_{v_{size}}^{2}+u_{v_{pres}}^{2}+u_{v_{speed}}^{2}.}$$ (25)

Using the typical values for uncertainty contributions for a fifth wheel, we approximate

$$u_{v_{5th}}\approx \sqrt{1.236\times 10^{-4}v_{5th}{}^{2}+4.075\times 10^{-3}\frac{m^{2}}{s^{2}}},\;\text{or}$$

1.1 km/h (0.68 mph) for *v* = 96.6 km/h (60 mph).

## 4. Uncertainty in Vehicle Speed, *u_v_*

The uncertainty in *v* as determined by a DTR radar will be primarily affected by the following: calibration method, *f*_0_, and clock frequency, as mentioned previously. Although other parameters and effects may contribute to speed measurement uncertainty, those uncertainty contributions are not observable given the limited resolution of DTR radar measurements. To compute *u_v_*, we need *v*, which we get by rearranging (3):

$$v=\frac{c\Delta f}{2f_{0}}.$$ (26)

The uncertainty can be written as:

$$u_{v}=\sqrt{v^{2}\frac{u_{f_{0}}^{2}}{f_{0}^{2}}+v^{2}\frac{u_{\Delta f}^{2}}{\Delta f^{2}}+u_{v,cal}^{2},}$$ (27)

where *u_v,cal_* is the uncertainty associated with one of the methods used to calibrate the DTR radar, that is, $u_{v,cal}=u_{v_{tf}}$, $u_{v_{AM}}$, $u_{v_{sm}}$, or $u_{v_{5th}}$.

The uncertainty in the DSP clock frequency, *f_clk_*, will affect the uncertainty in *Δf*. The value of *Δf* in current DTR radars is computed from the periodic sampling of the difference frequency output from the diode mixer.

The effect of *f_clk_* on *Δf* can be effectively understood by using:

$$\Delta f=rf_{clk},$$ (28)

where *r* is a constant that relates *f*_0_ and *f_clk_* for a given DTR radar. Therefore,

$$u_{\Delta f}=\sqrt{r^{2}u_{f_{clk}}^{2}+f_{clk}^{2}u_{r}^{2},}$$ (29)

where $u_{f_{clk}}\leq 10^{-5}f_{clk}$ and for typical clocks, with *f_clk_* ≈ 10 MHz, and Δ*f* ≤ 20 kHz, *r* ≤ 2 × 10^−3^. To be conservative, we set the uncertainty in *r*, *u_r_*, to be

$$u_{r}=\frac{u_{f_{0}}}{f_{0}}r,$$ (30)

which is about 10^−5^*r*. Putting all this together gives a value of *u_Δf_* of about 0.3 Hz.

Table 2 contains uncertainty values of parameters that contribute to speed measurement uncertainty. These are standard uncertainty values, computed for a target vehicle speed of 96.6 km/h (60 mph) and a confidence interval of 68.3 % (1*σ*). Only the calibration uncertainty corresponding to the calibration method used to calibrate the DTR radar should be used to compute speed measurement uncertainty.

**Fig. 3:**
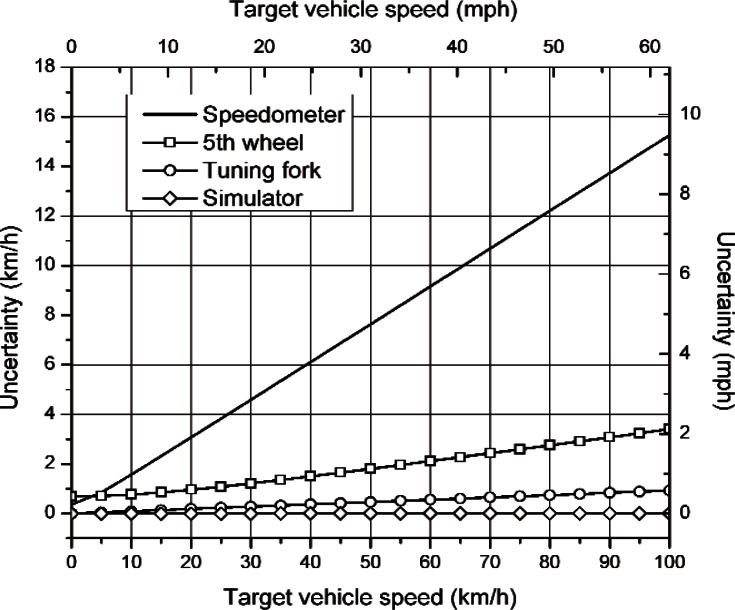
Uncertainty for each calibration technique for a 99.73 % confidence interval (3σ).

Table 3 lists the uncertainty in speed measurement (see Eq. 27) at several confidence intervals for each calibration method. Figure 3 shows a plot of these uncertainties for a 99.73 % confidence level (3*σ*) as a function of speed.

## 5. Conclusions

We have examined the uncertainty in vehicle speed measurements provided by the ubiquitous down-the-road (DTR) radar used by law enforcement. We included in this analysis the effects of the calibration (field performance verification) method, which uses one of the following tools: tuning fork, moving vehicle simulator, vehicle speedometer, or fifth wheel. This analysis does not consider uncertainties in speed measurement that are caused by operational issues with DTR radar usage.

The uncertainties in speed measurement due to calibration vary significantly depending on the method used to calibrate the DTR radar. The uncertainty in vehicle speed is the greatest using the vehicle’s speedometer as the calibration reference and the least when using the laboratory simulator. Furthermore, analysis provides information on the confidence to which a speed measurement can be given. The resulting equations from the uncertainty analysis are tabulated in Table 2. Table 3 and Fig. 3 are examples of how the resulting equations may be used to interpret the uncertainty for specific confidence intervals and speed values.

## Figures and Tables

**Table 1:** Parameters for the Tuning Forks used in this Study

Tuning fork	Frequency band	Design speed km/h (mph)	*S_TF_* (Hz/°C)	*f_TF_*_,0_ (Hz)
TF1	K	56.3 (35)	−0.688	2535.8
TF2	Ka	32.2 (20)	−0.552	2028.3
TF3	X	30 (18.6)	−0.152	589.4

**Table 2:** Uncertainty components and their values for a 68.3 % confidence interval (1*σ*) for a target vehicle speed of 96.6 km/h (60 mph)

Component	Value
$u_{v_{tf}}$	$3.1\times 10^{-3}v_{tf}$
$u_{v_{AM}}$	$1.4\times 10^{-5}v_{AM}$
$u_{v_{sm}}$	$\sqrt{2.587\times 10^{-3}v_{sm}{}^{2}+1.165\times 10^{-3}}$
$u_{v_{5th}}$	$\sqrt{1.236\times 10^{-4}v_{5th}{}^{2}+4.075\times 10^{-3}\frac{m^{2}}{s^{2}}}$
$u_{\Delta f}$	0.3 Hz
$u_{f_{0}}$	10^–5^*f*_0_

**Table 3:** Uncertainty in *v* for the various calibration methods and for various confidence intervals for a target vehicle speed of 96.6 km/h (60 mph)

Confidence interval	1*σ* (68.3 %) km/h [mph]	2*σ* (95.5 %) km/h [mph]	3*σ* (99.73 %) km/h [mph]	4*σ* (99.99 %) km/h [mph]	5*σ* (99.99994 %) km/h [mph]
Calibration method					
Vehicle Speedometer	4.9 [3.1]	9.8 [6.1]	15 [9.2]	20 [12]	25 [15]
Fifth Wheel	1.1 [0.68]	2.2 [1.4]	3.3 [2.0]	4.4 [2.7]	5.5 [3.4]
Tuning Fork	0.30 [0.19]	0.60 [0.37]	0.90 [0.56]	1.2 [0.74]	1.5 [0.93]
Simulator	2.2 × 10^−03^ [1.3 × 10^−03^]	4.3 × 10^−03^ [2.7 × 10^−03^]	6.5 × 10^−03^ [4.0 × 10^−03^]	8.6 × 10^−03^ [5.3 × 10^−03^]	1.1 × 10^−02^ [6.7 × 10^−03^]

## 7. Appendix A. Uncertainty Contributions to Tire Radius, $u_{r_{eff}}$

This appendix describes the various parameters that contribute to the uncertainty, $u_{r_{eff}}$, in the tire effective radius. All the formulas presented in this section, unless otherwise noted, are based on the study given in Ref. [4].

### A.1 Uncertainty of Tire Size, $u_{r_{size}}$

The change in *r_eff_* after speedometer calibration introduces an uncertainty component in *r_eff_* given by:

$$u_{r_{size}}=\left|\frac{r_{eff_{cal}}}{r_{eff}}-1\right|r_{eff}$$ (A1)

where $r_{eff_{cal}}$ represents the effective radii of the tires at the time of calibration and the vertical bars indicate absolute value.

### A.2 Uncertainty Due to Tire Brand or Type, $u_{r_{brand}}$

Tires of different construction may have different effective radii. It is not possible here to develop an equation accounting for all tire brands and type, but since tread depth and carcass thickness is fairly uniform across passenger car tires, the effect of a replacement tire of the same effective radius is expected to result in uncertainties in *r_eff_* of less than 1 % [4]. Consequently, we set

$$u_{r_{brand}=0.01r_{eff}}.$$ (A2)

### A.3 Uncertainty Due to Variation in Tire Inflation Pressure, $u_{r_{pres}}$

Changes in inflation pressure will be reflected as changes in the effective radius of the tire. The uncertainty in *r_eff_* associated with this pressure change is described by:

$$u_{r_{pres}}=\left|\frac{R_{nom}}{R_{nom}+\left(k_{P}\Delta P\right)}-1\right|r_{eff}$$ (A3)

where *R_nom_* is the nominal effective radius for the recommended inflation pressure, *k_P_* = 3.684 × 10^−6^ m/Pa (0.01 in/psi), and Δ*P* is the change in pressure from the recommended value [4].

### A.4 Uncertainty Due to Deviations From Normal Load, $u_{r_{load}}$

Changes in normal load, which describes the weight carried by the tire, will be reflected as changes in the effective radius of the tire. The uncertainty in *r_eff_* associated with this pressure change is described by:

$$u_{r_{load}}=\left|\frac{r_{nom}}{r_{nom}+\left(k_{load}\Delta F_{z}\right)}-1\right|r_{eff}$$ (A4)

where *r_nom_* is the nominal effective radius for a certain recommended load, *k_load_* is in the range 7.138 × 10^−7^ m/N to 8.157 × 10^−7^ m/N (1.250 × 10^−4^ in/lb to 1.429 × 10^−4^ in/lb), and Δ*F_z_* is the change in normal load from the recommended value [4].

### A.5 Uncertainty Due to Vehicle Speed, $u_{r_{speed}}$

The centrifugal forces on the tire vary with the speed of the vehicle and change the effective radius on the tire. The uncertainty in *r_eff_* associated with varying speed is described by:

$$u_{r_{speed}}=\left|\frac{R_{nom}}{R_{nom}+\left(k_{speed}\Delta v_{speed}\right)}-1\right|r_{eff}$$ (A5)

where *R_nom_* is the nominal effective radius at a certain vehicle speed, *k_speed_* = 5.682 × 10^−2^ s (1.250 × 10^−3^ in/mph), and Δ*v_speed_* is the change in vehicle speed from the nominal value [4].

### A.6 Uncertainty Due to Slip of the Tire on the Road, $u_{v_{slip}}$

Slip ratio is a measure of how fast the tire is rotating relative to how fast the road is passing beneath the tire. When the tire is free-rolling, that is, when there are no driving or braking forces, the slip ratio is zero. However, driving and braking forces both cause a slip ratio to develop which results in a change in *r_eff_*. The uncertainty in *r_eff_* associated with slip ratio is described by:

$$u_{r_{slip}}=\left|R_{slip}\right|r_{eff},$$ (A6)

where *R_slip_* is the slip ratio, in units of speed slip to target speed. Peak driving or braking forces on passenger car tires are associated with a slip ratio of ± 0.2, although most driving results in slip ratios ± 0.05. Similarly, spinning tires can cause speedometer reading much higher than the actual speed of the vehicle [4].

### A.7 Uncertainty Due to Tire Wear, $u_{r_{wear}}$

As a tire wears and the amount of tread diminishes, the effective radius of the tire decreases, and the exact amount of this decrease depends on the original tread depth and the internal construction of the tire. It is estimated that a 3.175 mm (0.125 in) loss in tread would result in less than 1 % error in *r_eff_* [4]. Consequently, we set

$$u_{r_{wear}}=0.01r_{eff}.$$ (A7)

There are other conditions; road grade, road surface, and vehicle cornering; which may effect speedometer measurements, but these are not likely to be encountered in a field calibration of the DTR radar and therefore are not explained further in this document.

### A.8 Uncertainty in *r_eff_*, $u_{r_{eff}}$

The uncertainty in the *r_eff_* from all the causes described previously in this section can be added together to give a value of $u_{r_{eff}}$:

$$u_{r_{eff}}=\sqrt{u_{r_{size}}^{2}+u_{r_{brand}}^{2}+u_{r_{pres}}^{2}+u_{r_{load}}^{2}+u_{r_{speed}}^{2}+u_{r_{slip}}^{2}+u_{r_{wear}}^{2}}.$$ (A8)

For typical values of the contributing uncertainty parameters, $u_{r_{eff}}$ ≈ 1.65 cm (0.65 in).
